# Response to Conway et al. (2023) from mothers and grandmothers: anti-industry bias, not formula marketing, is hurting us

**DOI:** 10.1017/S1368980023001040

**Published:** 2023-08

**Authors:** Ruth Ann Harpur, Arianna Andreangeli, Pamela Burns, Kathleen Cairns, Alieshia Cross, Susanna Haddon, Annie Hickox, Alice MacFarlane, Catherine Roy

**Affiliations:** Independent Scholar, United Kingdom of Great Britain and Northern Ireland

## Dear Editor

We write as mothers and grandmothers with lived experiences of the issues raised in the recent paper ‘Content analysis of on-package formula labelling in Great Britain: use of marketing messages on infant, follow-on, growing-up and specialist formula’^(1)^.

The paper examines compliance with Department of Health and Social Care (DHSC) legislation on formula marketing. Justification for this legislation hinges on the conviction that parents’ decisions about how to feed their babies are unduly influenced by formula marketing. The authors cite two pieces of evidence to back up this claim in their paper: firstly, an editorial outlining the financial value of the formula industry and its increasing presence in low-income settings^(2)^; secondly, an online survey, which found an association between agreement with common marketing claims and formula use in American mothers of babies over 6 months old and toddlers^(3)^.

It is unclear to us that the value of the formula industry is necessarily a reflection of undue influence on parent’s decisions. We are also confused as to how the opinions of mothers of older babies and toddlers are good evidence that marketing claims lead to the introduction of formula or the premature cessation of breastfeeding, as these most frequently occur in the first 6 weeks of a baby’s life^(4)^. Nor is it clear to us how a study conducted in a country which has relatively few restrictions on formula marketing, and which does not have a nationalised health service, would be applicable in a UK context.

In a press release, one of the authors states that the US boasts an exclusive breastfeeding rate of 19 % at 6 months, compared with 1 % in the UK^(5)^. But the USA did not sign up to the WHO code marketing restrictions and exerts relatively little regulation over formula marketing. This throws into question the authors’ conviction that marketing restrictions increase breastfeeding rates.

The reasons mothers give for stopping breastfeeding most commonly include pain, latching difficulties and low milk supply^(4)^. Qualitative studies in the UK reveal mothers in ‘agony’ and ‘constant’ pain, desperate to give up breastfeeding but feeling like they have to continue^(6)^. Studies also demonstrate guilt, shame and stigma associated with formula feeding in the UK^(7,8)^.

It is difficult for us to imagine that pictures on formula packaging of toys or baby animals can compete with the influence of breastfeeding promotion by trusted healthcare providers and public authorities. Additionally, we were simultaneously amused and appalled at the authors’ conclusion that while formula companies were 100 % compliant with the requirement to have a message that ‘breastfeeding is superior’ on every packet, the message was not prominent enough. One gets the impression that industry, like mothers, can do nothing right.

Parents who formula feed typically prepare formula multiple times a day. We wonder if the authors considered potential detrimental emotional effects on parents of seeing a prominent message that ‘breastfeeding is superior’ at each feeding, multiple times a day, every day, throughout their baby’s first year.

The authors cite no clear evidence that formula marketing unduly influences parents’ decisions. Nor do they cite any evidence that restrictions and requirements, such as insisting on a ‘breastfeeding is superior’ message, help women or babies to breastfeed. The evidence^(6–8)^ and our personal experience demonstrates that UK parents agonise over deciding to introduce formula or to stop breastfeeding, even when suffering intractably painful or stressful breastfeeding experiences. The narrative that our decisions are being unduly influenced by formula marketing comes across as insulting to parents, who have often made an unnecessarily difficult decision to formula feed in the face of considerable pressure to breastfeed from healthcare providers and broader parenting culture. It seems highly plausible that increasing restrictions might serve to exacerbate shame and stigma for families who formula feed.

The authors may not have intended their paper to be a commentary on parents’ decisions or experiences. However, the political and legislative context inevitably impacts us. It is surely, then, an oversight that in their desire to protect us from the perceived impact of formula marketing, they make no effort to reflect on the potential adverse effects of current regulations and the strengthening of restrictions that they recommend.

Is the goal really to protect parents, or is it a moral judgement about the formula industry and justification for punitive legislation? This may or may not ultimately harm formula companies, but it most certainly harms us. We are reminded of Hilda Bastian’s blog^(9)^ on pro- and anti-industry bias:

‘I think the main thing I learned – very painfully – in 20 years as a health consumer advocate, is that zealots always, always end up hurting patients. Because whatever it is that they are against, is not the same as being *for* patients, and it will, inevitably, betray us.’

When it comes to infant feeding policy, as parents, we wish to say very clearly: the anti-industry bias that currently pervades science and healthcare is betraying us.
